# Evaluation of Antibacterial Effects of Matrix-Induced Silver Ions against Antibiotic-Resistant ESKAPE Pathogens

**DOI:** 10.3390/ph14111094

**Published:** 2021-10-28

**Authors:** Ya-Chi Huang, Tsung-Ying Yang, Bo-Xuan Chen, Jung-Chang Kung, Chi-Jen Shih

**Affiliations:** 1 Department of Fragrance and Cosmetic Science, College of Pharmacy, Kaohsiung Medical University, Kaohsiung 807, Taiwan; yachi199918@gmail.com; 2 Department of Medical Laboratory Science and Biotechnology, College of Health Sciences, Kaohsiung Medical University, Kaohsiung 807, Taiwan; zegma040899@gmail.com (T.-Y.Y.); aa0970507086@gmail.com (B.-X.C.); 3 Center for Liquid Biopsy and Cohort Research, Kaohsiung Medical University, Kaohsiung 807, Taiwan; 4 School of Dentistry, College of Dental Medicine, Kaohsiung Medical University, Kaohsiung 807, Taiwan; 5 Department of Dentistry, Division of Family Dentistry, Kaohsiung Medical University Hospital, Kaohsiung 807, Taiwan; 6 Department of Dentistry, Kaohsiung Municipal Ta-Tung Hospital, Kaohsiung 801, Taiwan; 7 Department of Medical Research, Kaohsiung Medical University Hospital, Kaohsiung 807, Taiwan

**Keywords:** silver-containing mesoporous bioactive glass, matrix induction, silver ion, antibacterial agent, methicillin resistance, vancomycin resistance, carbapenem resistance, ESKAPE pathogens

## Abstract

Recently, drug-resistant bacterial infections, especially ESKAPE pathogens (*Enterococcus faecium, Staphylococcus aureus, Klebsiella pneumoniae, Acinetobacter baumannii, Pseudomonas aeruginosa, and Enterobacter* spp.), have become a critical health issue worldwide, highlighting the emerging need for novel antibacterial agents. In this study, silver nanoparticles were extracted from silver-containing mesoporous bioactive glass (MBG-Ag) using four different matrixes, including water, phosphate buffer saline (PBS), tryptic soy broth (TSB), and taurine (Tau). The inductively coupled plasma-mass spectrometer (ICP-MS) results demonstrated that the silver concentration of Tau-Ag was the highest among the four matrixes. The Tau-Ag was also observed to have 87.35% silver ions in its X-ray photoelectron spectrometer (XPS) spectra. The micrograph of transmission electron microscope (TEM) displayed a uniform distribution of silver nanoparticles, which was confined in a smaller size compared to that in TSB-Ag. Moreover, the peak shifts observed in the Fourier-transform infrared spectrometer (FTIR) spectrum implied that the -SO_3_^2−^ and -NH groups in taurine may interact with silver. A low cytotoxicity was noted for Tau-Ag, with approximately 70% of cells surviving at 0.63 mg/mL. Compared to the other three matrix-induced silver agents, Tau-Ag represented a better antibacterial effect against methicillin-resistant *Staphylococcus aureus*, with a minimum inhibitory concentration (MIC) value of 0.63 mg/mL and a postponed growth of 0.31 mg/mL observed. Further antibacterial examinations illustrated the presence of remarkable antibacterial activities against vancomycin-resistant *Enterococcus feacium*, carbapenem-resistant *Klebsiella pneumoniae*, carbapenem-resistant *Acinetobacter baumannii*, and carbapenem-resistant *Pseudomonas aeruginosa*. Given our observations and multiple bioactive functions of taurine (prevent patients from inflammation and oxidative-stress injuries), we anticipate that taurine matrix-induced silver ions would be a biomedical material with a high potential for combatting drug-resistant ESKAPE pathogens.

## 1. Introduction

Due to the overconsumption of antibiotics, the prevalence of antibiotic resistance has risen rapidly, especially in ESKAPE pathogens (*Enterococcus faecium*, *Staphylococcus aureus*, *Klebsiella pneumoniae*, *Acinetobacter baumannii*, *Pseudomonas aeruginosa*, and *Enterobacter* spp.), causing a serious threat to public health [1,2]. Vancomycin has been considered to be a last-resort antibiotic for treating antibiotic-resistant Gram-positive bacterial infections. However, vancomycin resistance in *Enterococcus* spp., particularly vancomycin-resistant *E. feacium* (VRE), has risen, causing a dilemma in clinical settings [3]. Methicillin-resistant *S. aureus* (MRSA) is a well-known antibiotic-resistant and Gram-positive pathogen discovered in the past century and is the major pathogen involved in skin and soft tissue infections, surgical infections, and orthopedic infections [4]. Owing to the increasing rate of methicillin resistance, alternative antibiotics have been widely used to treat MRSA infections, leading to a higher level of multidrug resistance in MRSA and limiting the available antibiotics. Carbapenem has been regarded as one of the last-line antibiotics for treating Gram-negative bacterial infections, including pneumonia, urinary tract infections, and bacteremia [5]. However, the extensive usage of carbapenem has triggered an increase in the rate of carbapenem resistance, limiting the treatment options for Gram-negative bacterial infections, such as carbapenem-resistant *K. pneumoniae* (CRKP), carbapenem-resistant *A. baumannii* (CRAB), and carbapenem-resistant *P. aeruginosa* (CRPA) [6,7,8]. To this end, the World Health Organization (WHO) has created a list of priorities for the development of novel antibacterial agents, including CRKP, CRAB, and CRPA, which are top priority, and VRE and MRSA, which are high priority [9], highlighting the emerging need for novel antibacterial agents.

Recently, much work has been carried out to document the antibacterial properties of metals in the biomedical field, such as silver, copper, and zinc [10]. Given its antimicrobial activity and stability, silver has been reported to be one of the most significant metals for antibacterial purposes [11]. The antibacterial activity of silver is attributed to the release of silver ions, which can bind to the thiol groups on bacterial proteins and further lead to DNA degradation and metabolic dysfunction (including respiratory reaction, lipid synthesis, and protein synthesis) [12]. In contrast to antibiotics, silver ions directly eliminate bacterial cells either chemically or physically [13,14]. Therefore, selection for strains resistant to silver antibacterial agents would occur less than for antibiotics. The functional materials of mesoporous bioactive glass (MBG) have received a great amount of attention in the biomedical field. These are usually synthesized by the evaporation-induced self-assembly (EISA) and sol-gel methods, which can obtain MBG with a uniform pore size and large surface area. In view of MBG’s characterizations, it could be applied for the controlled releasing and stabilization of functional contents [15,16,17], as well as for enhancing bone formation by inducing hydroxyapatite (HA) crystallization on its surface [18,19,20]. In previous studies, silver-containing mesoporous bioactive glass (MBG-Ag) has been used as a biomaterial for combatting multidrug-resistant bacteria. Additionally, the effects of the synthesis parameters and glass stoichiometry—such as the structure, composition, preparing temperature, surfactant type, silver concentration, and self-assembly time—on MBG-Ag have been studied in various works [21,22,23].

In a previous study, tryptic soy broth (TSB), a type of bacterial culture medium composed of protein, was used to extract silver from an MBG-Ag material via some kind of affinity between –SH and –NH groups in the TSB and silver [24]. The antibacterial activity of the extracted silver nanoparticles was 5 mg/mL, at which about 70% NIH3T3 cell survived, showing a low toxicity. Another study indicated that proteins in the TSB would reduce silver ions in the formation of silver nanoparticles (AgNPs) in the solution, and in the reducing process, the structure of the protein would change, thus doping the AgNPs [25]. The AgNPs increased in size with increasing reaction time, and the plasmon intensity at the reaction time of 11 h is near to that at 15 h. Even though the silver ions releasing from MBG-Ag in taurine was seldom discussed, there were some efforts on the interaction between silver and taurine or its derivative [26,27,28]. Taurine or its derivatives can form a chelate complex with metal ions, applying recycled metal ions from a water source. In particular, the complex of taurine and silver ions is more stable than the one of taurine and other metals. Also, taurine demonstrates extensive physiological activities within the body and presents in the retina, brain, heart, and placenta. Taurine contributes to bile acid conjugation, anti-inflammatory, maintenance of calcium homeostasis, osmoregulation, and membrane stabilization, and it was reported to have a protective effect against acute cardiovascular events [29,30]. Remarkably, taurine can prevent inflammation and slow down oxidative stress-mediated injuries [31]. A previous study indicated anti-atherogenic and anti-inflammatory effects for two weeks oral-administered taurine in heart failure patients. In our research, mediums used to induce silver from MBG-Ag were designated as matrices. Here, we sought to evaluate the release of Ag ions from different matrixes (water, phosphate buffer saline (PBS), TSB, and taurine) and their antibacterial capabilities against antibiotic-resistant ESAKPE pathogens. We expected to develop an antibacterial and anti-inflammatory agent.

## 2. Results and Discussion

### 2.1. The Potency of Matrixes to Induce Silver Ions

**Figure 1:**
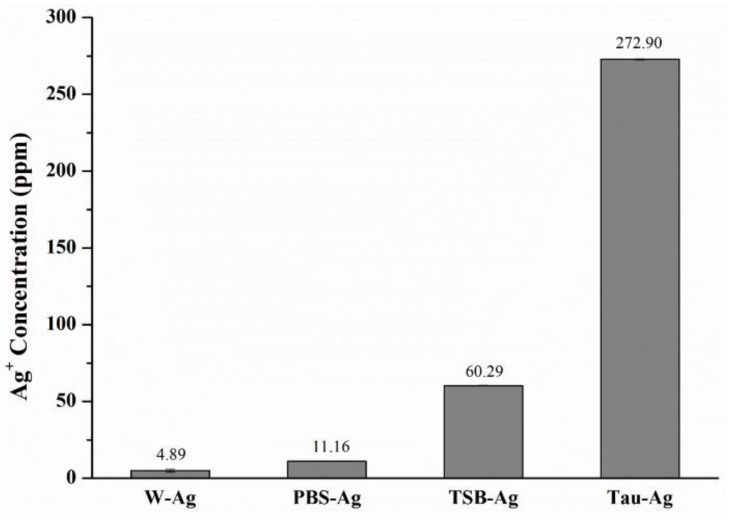
ICP-MS results of the silver content in W-Ag, PBS-Ag, TSB-Ag, and Tau-Ag.

As shown in Figure 1, the silver contents of four different matrixes were profiled using ICP-MS analysis. The silver content of water-induced silver (W-Ag) was revealed to be 4.89 ppm, while that of PBS-Ag was 11.16 ppm. A higher silver content was found in TSB-Ag (60.29 ppm), whereas among the four matrixes, the silver content of 272.90 ppm for Tau-Ag was found to be the highest, suggesting that taurine inducing silver from MBG-Ag had a better potency. In a previous study, B.W. Stuart et al. released silver ions from phosphate bioactive glasses in water, and the concentration of silver ions dramatically increased in 4 h, but slowed between 4 h and 12 h [32].

**Figure 2:**
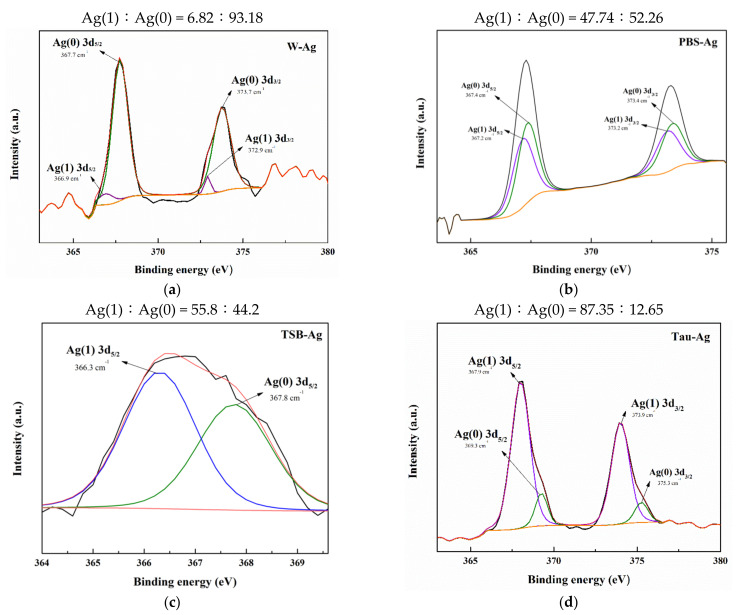
XPS results of (**a**) W-Ag, (**b**) PBS-Ag, (**c**) TSB-Ag, and (**d**) Tau-Ag patterns.

To better investigate the ion state of silver species in four different matrixes, the high-resolution XPS spectra of W-Ag, Tau-Ag, PBS-Ag, and TSB-Ag are demonstrated in Figure 2. The silver states were described as Ag(1) and Ag(0) for the ionic and metallic silver, respectively. The XPS spectra for Ag 3d_5/2_ and Ag 3d_3/2_ in W-Ag were revealed as Ag(0) (367.7 and 373.7 eV) and Ag(1) (366.9 and 372.9 eV) in Figure 2a; the Ag 3d_5/2_ and Ag 3d_3/2_ spectra for PBS-Ag were found to be Ag(0) (367.4 and 373.4 eV) and Ag(1) (367.2 and 373.2 eV) in Figure 2b; the Ag 3d_5/2_ and Ag 3d_3/2_ spectra for TSB-Ag were found to be Ag(0) (367.8 eV) and Ag(1) (366.3 eV) in Figure 2c; and the Tau-Ag spectrum was assigned to Ag(0) (369.3 and 375.3 eV) and Ag(1) (367.9 and 373.9 eV) in Figure 2d. Moreover, the ratio of Ag(1)/Ag(0) was found to be 0.07 in W-Ag, 0.91 in PBS-Ag, 1.26 in TSB-Ag, and 6.91 in Tau-Ag, illustrating a higher proportion of Ag(1) in Tau-Ag than in the other three matrixes. It is speculated that the proteins in TSB contained thiol groups and amine groups, which have a certain affinity for Ag [33]. Taurine (2-aminoethane sulfonic acid; ^+^NH_3_-CH_2_- CH_2_-SO_3_^−^) is an organic compound derived from cysteine; it is a conditionally essential amino acid and is found free or in simple peptides. Taurine has been reported by various researchers to interact with silver; moreover, taurine has been applied for recycling the silver ions from water resources and reducing the toxicity of metal [34,35]. Of many previous mechanistic studies, silver ions have been reported as the major culprit for the antibacterial activity of silver nanoparticle [36,37,38]. Silver ions could penetrate bacterial cells, condense DNA molecules, limit the cell replication, and bind to thiol groups and amino groups of protein, causing a decrease or loss of activity of multiple enzymes in bacteria. In our work, silver ions were significantly observed in Tau-Ag. For the above reasons, taurine is a medium with a high potential for inducing silver ions from MBG-Ag.

**Figure 3:**
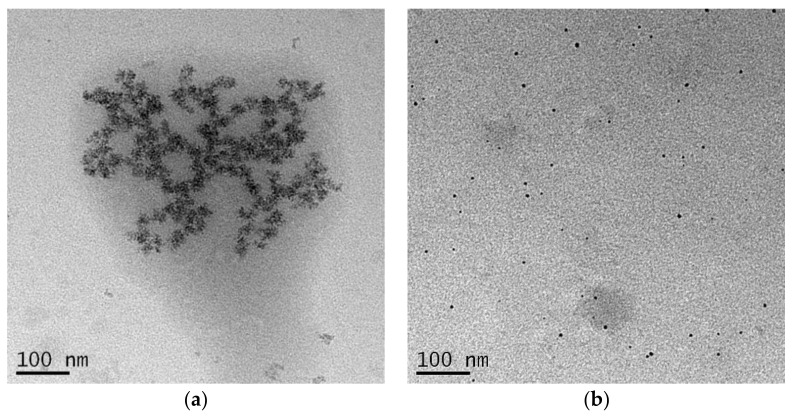
TEM images of (**a**) TSB-Ag and (**b**) Tau-Ag. Micrographs were captured at 25,000× magnification.

TSB-Ag and Tau-Ag, two matrixes possessing higher contents of silver ions, were further observed by TEM (Figure 3). For TSB-Ag, it was shown that larger AgNPs were coalesced and incorporated in the structure of the protein (Figure 3a). By contract, Figure 3b shows that the AgNPs in Tau-Ag were present in a low content, were uniformly distributed, and had smaller sizes. No observable particle was seen in the micrographs of W-Ag and PBS-Ag (data not shown). Given the observations of Tau-Ag, a higher surface area could be expected, which would lead to more partial oxidation of silver and result in a higher release of silver ions. The observation of the low content of AgNPs also supported the result from the XPS spectra of Tau-Ag, which showed a high Ag(1)/Ag(0) ratio.

**Figure 4:**
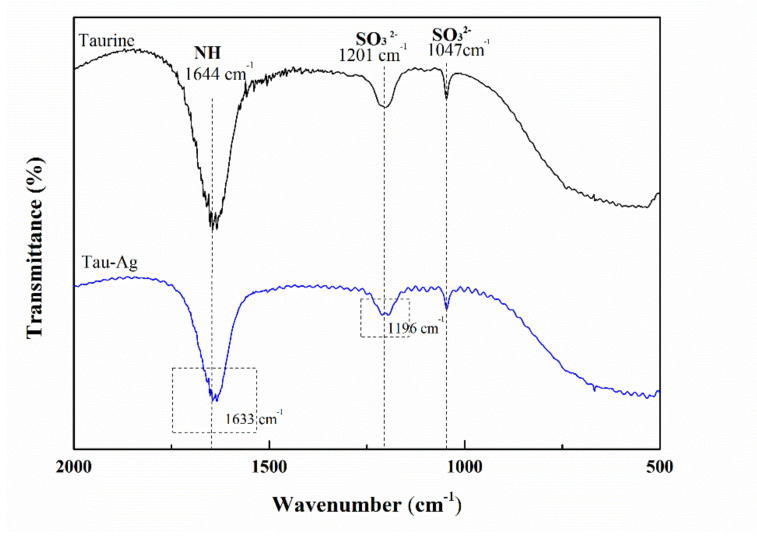
FTIR spectra of taurine and Tau-Ag.

To further understand the release of Ag ions in taurine, FTIR was used to analyze the frequency shift of Tau-Ag. The frequency shifts of sulfonic acid (SO_3_^2−^) and the NH_2_ vibration of the ligand from 1201 and 1644 cm^−1^ to lower frequencies (Figure 4) confirmed its involvement in co-ordination [33]. The -NH and -SO_3_^2−^ groups of taurine might have some affinity with silver [33], while the percentages of unbound Ag, Ag–O, Ag–N, and Ag–S were found to be 2, 71, 11, and 16, respectively [39]. It could be expected that the SO_3_^2−^ and NH_2_ groups may have some level of affinity with silver.

### 2.2. Cytotoxicity of Tau-Ag

**Figure 5:**
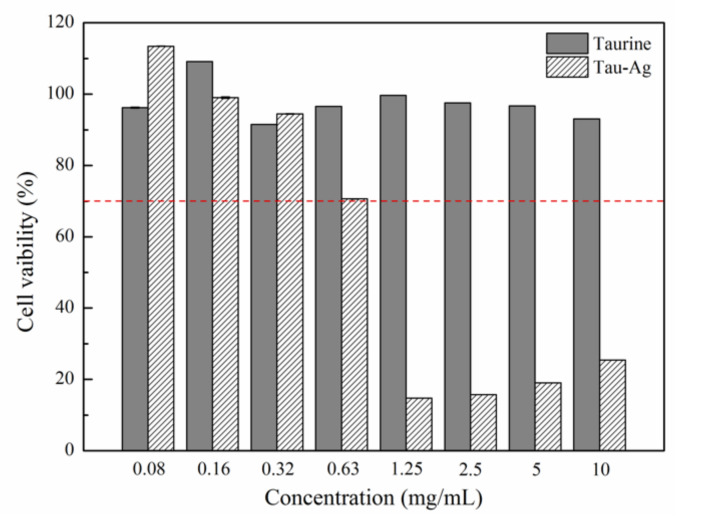
MTS assay result for NIH-3T3 treated with different concentrations of Tau-Ag. The results are expressed as the percentage of cell viability relative to the control. The values represent the averages of three independent experiments with triplicate measurements (mean ± SD). The red dotted line indicated 70% cell viability for 0.63 mg/mL of Tau-Ag.

The matrix-induced silver ions, Tau-Ag, were observed to have the highest ability to release silver ions among the four different matrixes. Thus, we examined the cytotoxicity of Tau-Ag against NIH-3T3 fibroblast cells to discover the feasibility of its clinical application, adhering to the American National Standard ISO 10993-5. Cells were treated with different concentrations of taurine and Tau-Ag (0.08, 0.16, 0.32, 0.63, 1.25, 2.5, 5, 10 mg/mL) for 24 h. Cell viability was determined using the MTS assay. Nearly 100% cell viabilities were noticed for Tau-Ag in concentrations of 0.08, 0.16, and 0.32 mg/mL (Figure 5), while an approximately 70% cell viability was found for 0.63 mg/mL of Tau-Ag, indicating the biocompatibility of Tau-Ag.

### 2.3. Tau-Ag Combats Antibiotic-Resistant ESKAPE Pathogens

**Figure 6:**
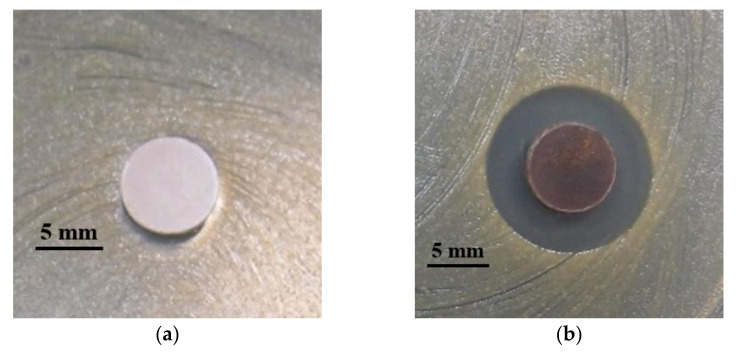
Quality control testing for (**a**) MBG and (**b**) MBG-Ag against MRSA using a disk diffusion method.

The MBG (Figure 6a) did not exhibit antibacterial properties, whereas Figure 6b represented the inhibition zone of MGB-Ag against MRSA after a 24 h incubation period (inhibition zone = 15 mm). This result demonstrated the successful synthesis of MBG-Ag.

**Figure 7:**
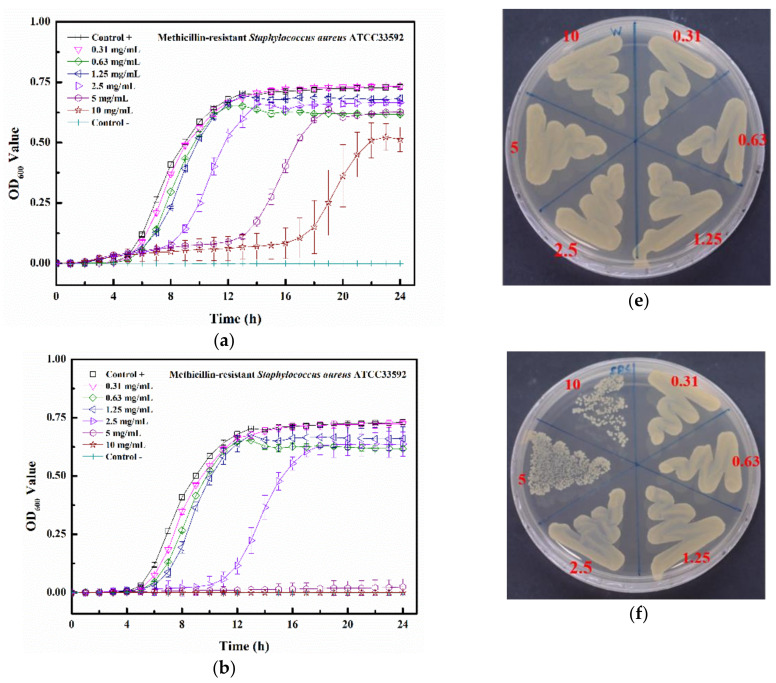

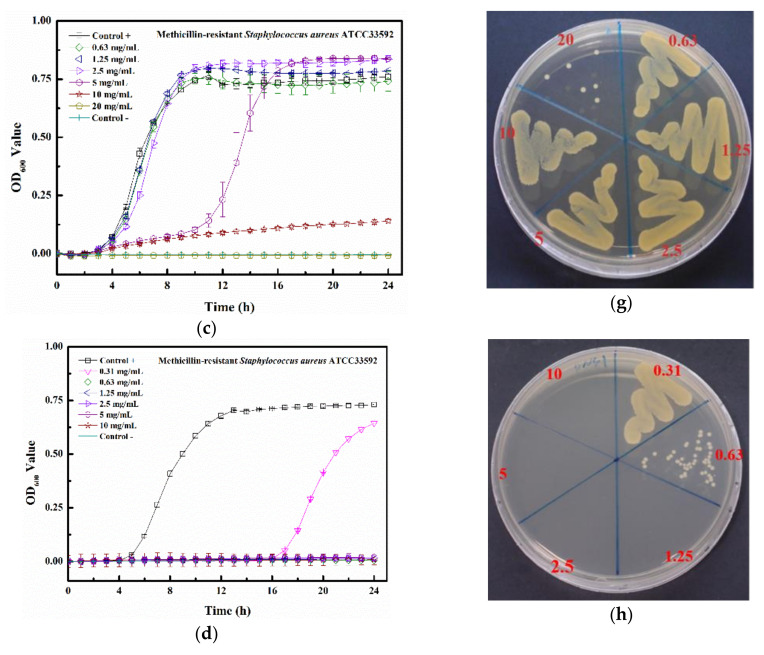
Growth curve results for (**a**) W-Ag, (**b**) PBS-Ag, (**c**) TSB-Ag, and (**d**) Tau-Ag. The results are represented as the absorbance at 600 nm, with dots as means and bars as SD. Colony-forming tests results for (**e**) W-Ag, (**f**) PBS-Ag, (**g**) TSB-Ag, and (**h**) Tau-Ag.

The kinetic bacterial growth assays of four matrix-induced silver ions at different concentrations were monitored each hour (up to 24 h) (Figure 7a–d). The water-induced silver possessed only a low inhibitory activity against MRSA ATCC 33592, with delayed bacterial growths observed at 2.5, 5, and 10 mg/mL. The minimum inhibitory concentration (MIC) values of PBS-Ag, TSB-Ag, and Tau-Ag against MRSA ATCC 33592 were 5, 10, and 0.63 mg/mL (Figure 7b–d), respectively, and the postponed bacterial growths were noted at the sub-MIC level (concentrations two-fold lower than the MIC). Both the sub-MICs of PBS-Ag (2.5 mg/mL) and TSB-Ag (10 mg/mL) lowered the bacterial growth from 4 to 12 h (Figure 7b,c), while the postponed bacterial growth in the sub-MIC of Tau-Ag (0.31 mg/mL) recommenced at 16 h (Figure 7d). A colony-forming assay demonstrated that there was no decrease in colonies for W-Ag up to 10 mg/mL (Figure 7e), suggesting the poor activity of W-Ag. Slight reductions in the amounts of bacteria were noticed at 5 and 10 mg/mL of PBS-Ag (Figure 7f), whereas an observable decrease was noted at 20 mg/mL of TSB-Ag (Figure 7g). Remarkable changes were found for Tau-Ag at 0.63 and 1.25 mg/mL (Figure 7h), even with no colony presenting on agar at 1.25 mg/mL. Tau-Ag, which possessed the most robust antibacterial activity, was chosen for further analysis.

**Figure 8:**
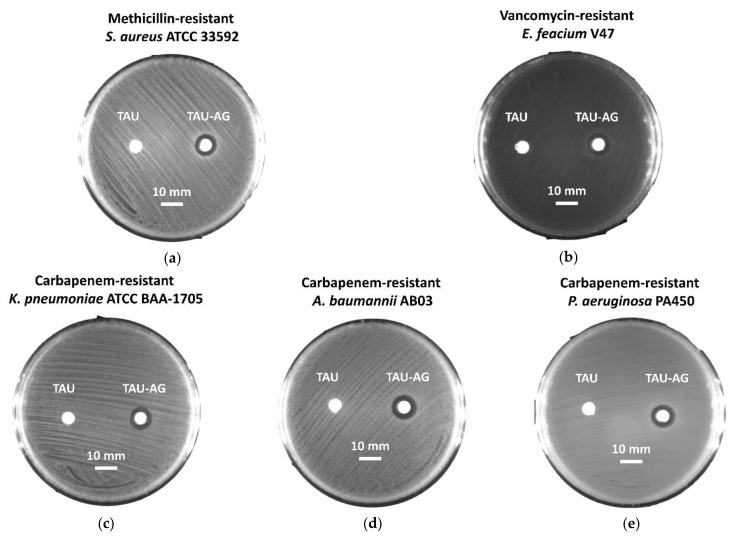
Disk diffusion analysis for (**a**) methicillin-resistant *S. aureus* (MRSA) ATCC 33592, (**b**) vancomycin-resistant *E. feacium* (VRE) clinical isolate V47, (**c**) carbapenem-resistant *K. pneumoniae* (CRKP) ATCC BAA-1705, (**d**) carbapenem-resistant *A. baumannii* (CRAB) clinical isolate AB03, and (**e**) carbapenem-resistant *P. aeruginosa* (CRPA) clinical isolate PA450. The scale represents a 10 mm length. The Tau disks contain 10 μL of 2% taurine solution each as a control, while the disks of Tau-Ag were dipped in 10 μL of taurine-induced silver ions (20 mg/mL).

As suggested by the results of the growth curve and colony-forming testing, an observable inhibition zone was noticed for MRSA ATCC 33592, with a diameter of 10 mm (Figure 8a). An antibacterial effect was found for VRE strain V47, with an inhibition zone of 11 mm (Figure 8b). Furthermore, the inhibitory activities of Tau-Ag were also demonstrated for carbapenem-resistant strains, including CRKP with a 10 mm inhibition zone (Figure 8c), CRAB with an 11 mm one (Figure 8d), and CRPA with a 10 mm one (Figure 8e). In view of the results from the disk diffusion assays, the kinetic antibacterial activities and colony-forming analyses of Tau-Ag against VRE, CRKP, CRAB, and CRPA were further examined.

**Figure 9:**
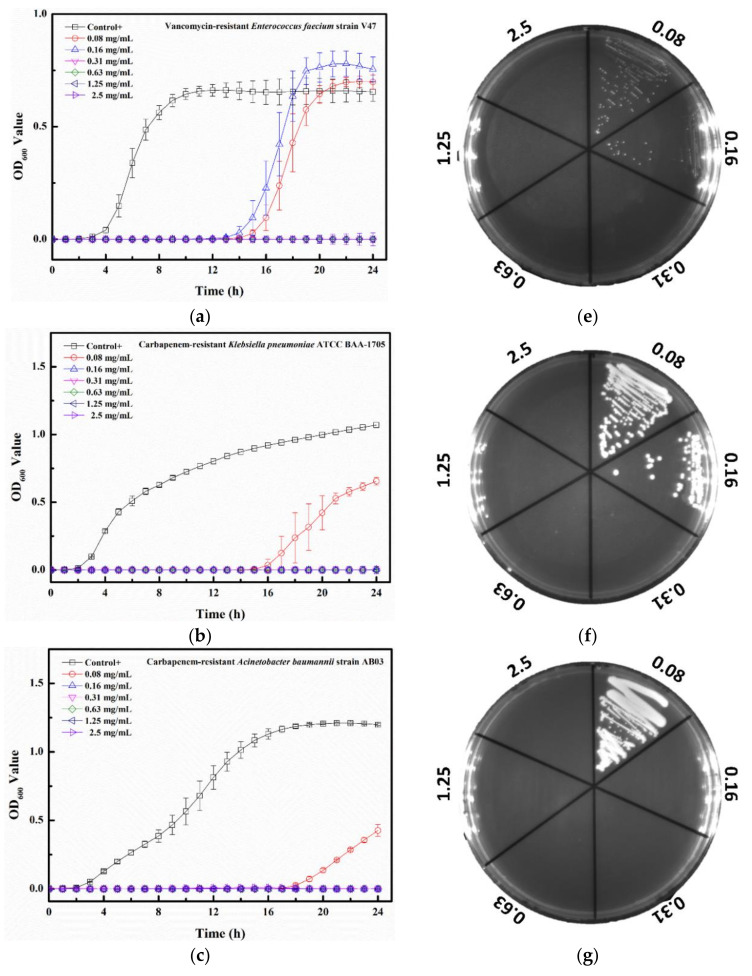

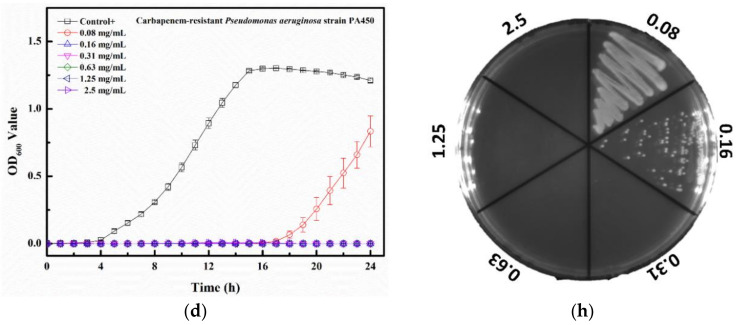
Growth curve results for Tau-Ag against (**a**) vancomycin-resistant *E. feacium* (VRE) clinical isolate V47, (**b**) carbapenem-resistant *K. pneumoniae* (CRKP) ATCC BAA-1705, (**c**) carbapenem-resistant *A. baumannii* (CRAB) clinical isolate AB03, and (**d**) carbapenem-resistant *P. aeruginosa* (CRPA) clinical isolate PA450. The results represent absorbance at 600 nm, with dots as means and bars as SD. Colony-forming tests results for (**e**) VRE V47, (**f**) CRKP ATCC BAA-1705, (**g**) CRAB AB03, and (**h**) CRPA PA450.

The MIC value of Tau-Ag against VRE V47 was 0.31 mg/mL (Figure 9a), with postponed growths found at both 0.08 and 0.16 mg/mL. Similar antibacterial effects were observed for CRKP, CRAB, and CRPA, demonstrating that the MIC values were all 0.16 mg/mL, and delayed growths were found at 0.08 mg/mL for all bacterial strains (Figure 9b–d). The colony-forming assays revealed that the minimum bactericidal concentration (MBC) values for VRE, CRKP, and CRPA were all 0.31 mg/mL, all with dose-dependent reductions in colonies at concentrations from 0.08 to 0.16 mg/mL (Figure 9e,f,h), whereas the MBC value for CRAB was noticed at 0.16 mg/mL (Figure 9g). Silver ions have been documented to be one of the major antibacterial mechanisms of silver; thus, the release of silver ions would significantly affect the antibacterial properties of silver [40]. In a previous study, platinum (Pt) was utilized to enhance the silver ion formation through galvanic actions [41]. The antibacterial activities were examined by coating silver on polyurethane with or without Pt, and the results demonstrated that compared to the silver without Pt, silver with Pt eliminated 2-log more bacteria on the surface, showing a boost in the antibacterial activity caused by Pt. In a comparative study, the antibacterial effects of silver ions and silver nanoparticles with two different diameters (5 and 20 nm) were evaluated [42]. Silver ions were observed to have 2- to 8-fold lower MIC values than the two silver nanoparticles, suggesting that the antibacterial potency of silver ions was higher. Moreover, decreased plasma taurine has been linked to many diseases, especially inflammatory diseases [43,44], implying that supplementation with taurine might offer relief from infections to some degree. In our work, the matrix, taurine, was used to induce silver ions from a silver-containing mesoporous bioactive glass, and Tau-Ag was observed to have a high antibacterial activity against the antibiotic-resistant pathogen ESKAPE.

## 3. Materials and Methods

### 3.1. Synthesis of Silver-Containing Mesoporous Bioactive Glass (MBG-Ag)

MBG-Ag was synthesized using the Evaporation-Induced Self-Assembly (EISA) and sol-gel methods [45], with a 80SiO_2_-15CaO-5P_2_O_5_ composition doped with a 1 mole ratio of silver, which was performed as previously described. In short, precursors, including tetraethylorthosilicate (TEOS), triethyl phosphate (TEP), calcium nitrate (Ca(NO_3_)_2_⋅4H_2_O), and sliver nitrate (AgNO_3_), were added to 2 M of nitric acid (in ethanol). The non-ionic surfactant F127 was utilized as a structure-directing agent. A sol was formed via stirring the mixture at room temperature for 24 h. Polyurethane foam (PUF) was employed as a scaffold for the solution gelation, allowing each sol to be pressed into PUF pores. The scaffolds were dried at 100 °C, then PUF, F127, and acid groups in gels were removed by thermal treatment at 600 °C for 2 h. Moreover, according to our previous work [46], diffraction peaks at 38.1°, 44.3°, 64.5°, and 77.4° were observed in the X-ray diffraction (XRD) spectrum of the MBG-Ag, which is similar to the crystallization of silver (JCPD 87-0597). To confirm the antibacterial quality of the synthesized MBG-Ag, a disk diffusion assay was performed to test the effects against methicillin-resistant *S. aureus* (MRSA) ATCC 33592. Briefly, the powders of MBG and MBG-Ag were compressed into 8 mm disks and subsequently placed onto TSB agar with a bacterial lawn of MRSA ATCC 33592. After 24 h of incubation at 37 °C, clear zones around the disks were defined as the inhibition zones and the diameters were measured.

### 3.2. Preparation of Matrix-Induced Silver

MBG-Ag was used as the source of silver ions, while water, phosphate buffered saline (PBS; GeneDirex, Inc., Taipe, Taiwan), TSB (Acumedia, Neogen corporation, Lansing, MI, USA), and taurine (Acros Organics, Geel, Belgium) were used as matrixes to induce the release of silver ions from MBG-Ag. Briefly, 0.2 g of MBG-Ag powder was added to 10 mL of water, PBS, 3% TSB, or 2% taurine solutions, and then shaken at 160 rpm at 37 °C for 24 h to obtain 20 mg/mL (solid–liquid ratio) of matrix-induced silver ions. The resulting solution was filtered with a 0.22 μm filter to remove solid material, then the matrix-induced silver ions were designated as W-Ag (water), PBS-Ag (PBS), TSB-Ag (TSB), and Tau-Ag (taurine), respectively.

### 3.3. Characterization of Matrix-Induced Silver

The solutions of matrix-induced silver ions were subjected to quantify the silver-releasing ability of each matrix using an -ICP-MS (Thermo-Element XR, Thermo Fisher Scientific Inc., Bremen, Germany). An XPS analysis was carried out to determine the Ag(1)/Ag(0) ratio in four solutions of matrix-induced silver ions with a PHI 5000 VersaProbe Scanning XPS Microprobe (ULVAC-PHI, Kanagawa, Japan) equipped with the monochromatized Al K_α_ X-ray source (1486.6 eV, 15 kV, 10 mA). The binding energy was calibrated using reference C 1 s photoelectrons at 284.8 eV. Shirley background subtraction based on the Gaussian–Lorentz sum function was used to fit the spectra. All the spectra were analyzed using the spectra deconvolution software, CasaXPS. To analyze the protein-encapsulated silver in TSB-Ag, the PHI QuanteraII High Resolution X-ray Photoelectron Spectrometer (ULVAC-PHI, Kanagawa, Japan) was used, with GCIB sputtering at different accelerated voltages and cluster sizes and monatomic Ar^+^ sputtering at different accelerated voltages after each sputtering cycle.

### 3.4. Analysis of Tau-Ag Matrix-Induced Silver Ions

Micrographs of TSB-Ag and Tau-Ag were captured by TEM using a JEM-2100 electron microscope (Jeol Ltd., Tokyo, Japan) operating at 200 kV. Fourier-transform infrared spectroscopy (FTIR) spectra were recorded using a FTIR spectrometer (Thermo Nicolet 6700, Thermo Fisher Scientific Inc., Waltham, MA, USA) with a range of 500~2000 cm^−1^ to understand the frequency shift of the Tau-Ag which possessed the highest silver ion-releasing ability.

### 3.5. In Vitro Cytotoxicity Testing

An in vitro toxicity test was performed using an MTS assay with the CellTiter-96^®^ aqueous cell proliferation kit (Promega, Madison WI, USA). Based on the American National Standard ISO 10993-5 [47], NIH-3T3 fibroblast cells (5 × 10^4^) were plated in 96-well plates and treated with different concentrations of taurine and Tau-Ag (0.08, 0.16, 0.32, 0.63, 1.25, 2.5, 5, 10 mg/mL) in serum-free medium (DMEM) at 37 °C for 24 h. The cells of the control group were treated without any materials, but with the same procedure. After incubation, 20 μL of MTS solution was added to each well of the 96-well assay plate containing the samples in 100 μL of culture medium, then the cells were incubated at 37 °C for a further 2 h. The absorbance of the well contents was detected at 490 nm using a Multiskan Sky Microplate Spectrophotometer (Thermo Fisher Scientific, Waltham, MA, USA). Cell viability was calculated using the following equation:

$$\mathrm{Cell}\;\mathrm{viability}\;\left(\%\right)=\frac{A_{s}}{A_{c}}\times 100\%,$$

where *A*_c_ is the average of three replicate absorption values of the control group and *A*_s_ is the average of three replicate absorption values of the sample groups.

### 3.6. Bacterial Strains

Vancomycin-resistant *E. feacium* (VRE) clinical isolate V47, methicillin-resistant *S. aureus* (MRSA) ATCC 33592, carbapenem-resistant *K. pneumoniae* (CRKP) ATCC BAA-1705, carbapenem-resistant *A. baumannii* (CRAB) clinical isolate AB03, and carbapenem-resistant *P. aeruginosa* (CRPA) clinical isolate PA450 were employed for the further examination of Tau-Ag. All strains were frozen at −80 °C, waiting for examinations. Before assays, testing strains were recovered onto tryptic soy agar supplied with 5% sheep blood (blood agar plate, BAP; Creative Media Plate, New Taipei City, Taiwan) and incubated at 37 °C for 24 h. Subsequently, bacteria were sub-cultured and incubated at 37 °C for another 24 h to stabilize the physiological characterization.

### 3.7. Disk Diffusion Assay

The antibacterial activities of Tau-Ag against antibiotic-resistant ESKAPE pathogens were preliminarily investigated using the disk diffusion method. The empty disks were prepared via punching filter paper, then the 8 mm disks were subjected to autoclave and dried at 80 °C overnight. The testing bacterial strain was suspended in 0.01 M PBS at 0.5 McFarland (contained 2 × 10^8^ CFU/mL), and the suspension was spread onto tryptic soy agar using a swab. Two sterilized disks were attached to the agar plate with the bacterial lawn. A total of 10 μL of the matrix-induced silver ions, Tau-Ag at 20 mg/mL, was added to one of the disks, and 10 μL of 2% taurine solution was applied to the other disk as a control. Plates were incubated at 37 °C overnight, and the diameters of the inhibition zones were measured. The pictures in Figure 8 were taken using an imager system (SmartView Pro 2100 Imager System, CCD UVCI-2100, Major Science, Saratoga, CA, USA) with white lights.

### 3.8. Growth Curve Analysis

Growth curve analyses were performed as previously described, and MRSA ATCC 33592 was chosen for evaluating the antibacterial activities of silver ions induced by four different matrixes (W-Ag, PBS-Ag, TSB-Ag, and Tau-Ag). Briefly, the bacterial suspension of MRSA ATCC 33592 was adjusted to 0.5 McFarland (2 × 10^8^ CFU/mL) and diluted 200-fold in TSB. Silver ions induced by four different matrixes were diluted two-fold to concentrations ranging from 1.25 to 20 mg/mL. Totals of 100 μL of the diluted bacterial suspension and 100 μL of the matrix-induced silver ions were added and mixed in flat-bottom 96-well plates, with a final bacterial density of 5 × 10^5^ CFU/mL and a matrix-induced silver ion concentration ranging from 0.63 to 10 mg/mL. Bacterial growth was determined by measuring the optical density at 600 nm every hour (up to 24 h) at 37 °C using a spectrophotometer (Tecan, Switzerland). The control broth contained TSB medium and matrix-induced silver ions at different concentrations. After the validation of the antibacterial effect for Tau-Ag, the remaining bacterial strains (VRE, CRKP, CRAB, and CRPA) were only tested in Tau-Ag at concentrations ranging from 0.08 to 2.5 mg/mL. The MIC was defined as the minimum concentration that resulted in no visible growth after 24 h. All the experiments were carried out in triplicate.

### 3.9. Colony-Forming Assay

Cultures from growth curve analyses were harvested as samples for colony-forming assays. One loopful of 1 μL cultural sample was spread onto TSA plates using a standard loop (BD, Difco^TM^, Cat. No. 220215). The TSA plates were then incubated at 37 °C for 24 h. The MBC was determined as the lowest concentration of materials (mg/mL) that showed no appearance of any bacterial colony on the plate.

## 4. Conclusions

Taken together, our work demonstrated that silver ions induced by a matrix, taurine, possessed biocompatibility and highly antibacterial activities against antibiotic-resistant ESKAPE pathogens, with an MIC ranging from 0.16 to 0.31 mg/mL, which allowed over 70% of the NIH3T3 cells to survive. With multiple bioactive functions of taurine (prevent patients from inflammation and oxidative-stress injuries), the use of taurine in formulations might be a remarkable strategy to develop a novel antibacterial agent. Further investigations of the use of taurine-induced silver ions as an antibacterial agent are needed in future studies.

## Data Availability

Data is contained within the article.
